# An innovative web-based decision-aid about birth after cesarean for shared decision making in Taiwan: study protocol for a randomized control trial

**DOI:** 10.1186/s13063-023-07103-8

**Published:** 2023-02-09

**Authors:** Shu Wen Chen, Allison Shorten, Chang Ching Yeh, Chien Huei Kao, Yu Ying Lu, Hsiang Wei Hu

**Affiliations:** 1 School of Nursing, National Taipei University of Nursing and Health Sciences, Taipei, Taiwan; 2 School of Nursing, University of Alabama at Birmingham, Birmingham, USA; 3 Department of Obstetrics and Gynecology, Taipei Veterans General Hospital, Taipei, Taiwan; 4 Department of Nursing-Midwifery and Women Health, National Taipei University of Nursing and Health Sciences, Taipei, Taiwan; 5 Department of Biomedical Engineering, National Cheng Kung University, Tainan, Taiwan

**Keywords:** Shared decision making, Web-based decision-aid, Vaginal birth after caesarean, RCT

## Abstract

**Background:**

Taiwan has a high national caesarean rate coupled with a low vaginal birth after caesarean (VBAC) rate. This study aims to develop and evaluate a web-based decision-aid with communication support tools, to increase shared decision making (SDM) about birth after caesarean.

**Methods:**

A quantitative approach will be adopted using a randomized pre-test and post-test experimental design in a medical centre in northern Taiwan. The web-based decision aid consists of five sections. Section 1 provides a two-part video to introduce SDM and how to participate in SDM. Section 2 presents an overview of functions and features of the birth decision-aid. Section 3 presents relevant VBAC information, including definitions, benefits and risks, and an artificial intelligence (AI) calculator for rate and likelihood of VBAC success. Section 4 presents the information regarding elective repeat caesarean delivery (ERCD), involving definitions, benefits, and risks. Section 5 comprises four steps of decision making to meet women’s values and preferences. Pregnant women who have had one previous caesarean and are eligible for VBAC, will be recruited at 14–16 weeks. Participants will complete a baseline survey prior to random allocation to either the control group (usual care) or intervention group (usual care plus an AI-decision aid). A follow up survey at 35–38 weeks will measure change in decisional conflict, knowledge, birth mode preference, and decision-aid acceptability. Actual birth outcomes and satisfaction will be assessed one month after birth.

**Discussion:**

The innovative web-based decision-aid with support tools will help to promote pregnant women’s decision-making engagement and communication with their providers and improve opportunities for supportive communication about VBAC SDM in Taiwan. Linking web-based AI data analysis into the medical record will also be assessed for feasibility during implementation in clinical practice.

**Trial registration:**

ClinicalTrials.gov identifier (NCT05091944), Registered on October 24, 2021.

## Introduction

### Background and rationale {6a}

Elective repeat cesarean delivery (ERCD) contributes to a significant proportion of cesarean delivery (CD) rates in many high and middle-income countries [1–4]. Although CD is considered to be a relatively safe procedure, it has been associated with an increased risk of adverse maternal and neonatal outcomes [5, 6]. A recent scoping review showed that women are not adequately engaged in decision-making in relation to planned CD [7]. Vaginal birth after caesarean (VBAC) is a safe and reasonable alternative for most women who have experienced previous CD [8], yet many health professionals and health care organizations remain reluctant to offer VBAC, in part due to concerns about uterine rupture and perinatal death [9–12].

Shared decision making (SDM) is a patient-centered approach to promote collaboration between patients, family members, and healthcare providers as they navigate complex value-sensitive decisions together [7, 13–15]. SDM is the preferred approach for making healthcare decisions when more than one reasonable option is available [16]. Compared to the traditional paternalistic model of decision making, SDM emphasizes that the patient is actively involved in decision making, with information exchange and opportunity to express treatment preferences [17]. SDM is a process in which decisions are made in a collaborative way, where trustworthy evidence-based information is provided in accessible formats about a set of options, typically in situations where the concerns, personal circumstances, and contexts of patients, and their families play a major role in decisions [18]. There is a clear need for medicine to be more patient centered and for patients to be more actively involved in their care decisions [16]. Clinicians and patients need to work together to understand the patient’s situation, and to determine how best to address patient problems [16]. Effective communication is essential for achieving SDM [14, 16]. A three talk model of SDM involves choice talk, option talk and decision talk [18]. With further development, the three talk was modified into team talk, option talk and decision talk [18]. The revised three-talk model of SDM depicts conversational steps, initiated by providing support when introducing options, followed by strategies to compare and discuss trade-offs, before deliberation, based on informed preferences [18]. It is critical to develop an understanding of the human situation, to work together, to discuss and discern ways forward that make sense for each person in context [16]. Such humanistic practice is rooted in the recognition of the human meaning of care and in a concern for human emotions, source of suffering, and peace of mind [16]. Without empathy, the execution of the SDM process could be formally correct but ineffective in practice [16].

Taiwan has sustained a high national caesarean rate of over 35% for more than a decade [19]. Studies suggest that efforts to increase VBAC could be potentially important in reducing the high CD rates in Taiwan [20–22]. In 2003, the Bureau of National Health Insurance (NHI) used to propose a VBAC case payment to encourage physicians performing VBAC with the same case payment as CD in 2003. In 2005, NHI further proposed another policy of health insurance payment with the equivalent fees for a normal vaginal birth and CD [23–27]. However, the financial incentives, designed specifically for obstetricians, had limited effects on CD rates [24–26]. The prevalence of VBAC has remained less than 0.5% over two decades in Taiwan [19]. Several factors influence Taiwanese women’s decision making. Previous studies reveal that women have limited knowledge regarding birth choices after caesarean [28–30]. Lack of reliable information regarding the risks and benefits of VBAC resulted in women’s reluctance to attempt VBAC, due to fear of uterine rupture. Internet information, female friends, and family members were important sources of information and influential in women’s decision making about mode of birth [29, 31]. Chen et al. (2021) conducted a pilot study using an evidence-based decision-aid booklet to support women’s birth choices in Taiwan. Results demonstrated that using a birth choice decision-aid would be feasible to increase women’s knowledge, decrease decisional conflict and improve satisfaction [30]. However, despite these findings, the pilot study also revealed that many Taiwanese women were not prepared or expecting to participate in SDM with their obstetricians due to concerns about time constraints during consultations, and fear of asking questions [30].

### Objectives {7}

To redress gaps, developing a humanistic, women-centered decision support tool could potentially assist women, their families, and providers, to navigate value-laden birth decisions in Taiwan. Thus, this study aims to develop and evaluate a web-based decision-aid platform with communication tools to support women and providers in SDM about birth after cesarean in Taiwan.

### Trial design {8}

**Fig. 1:**
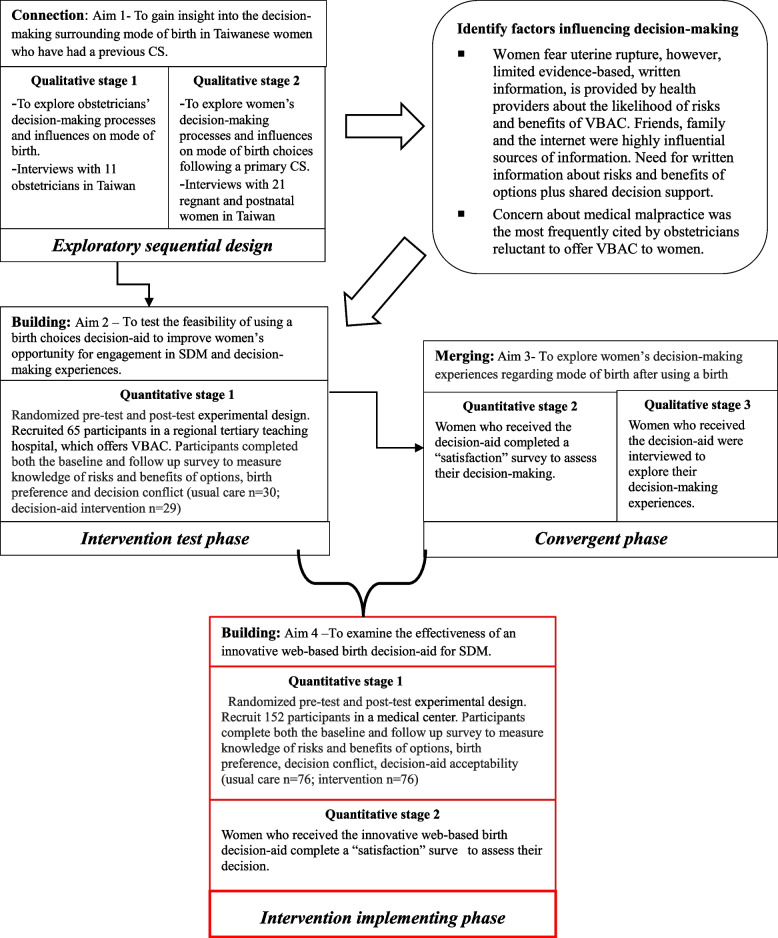
Study design

The Ottawa Decision Support Framework (ODSF) is utilized in the trial design [32]. Figure 1 outlines the larger multi-phase mixed methods study design sequence which the current study is nested into [17, 29, 30, 34]. The current study represents Aim 4 (Intervention implementing phase) (Fig. 1) and is a randomized control trial design. Stage 1 involves a randomized pre-test and post-test experimental design; decisional conflict, knowledge, birth mode preference, and decision-aid acceptability will be assessed. Stage 2 comprises a survey to measure actual birth outcome (VBAC or ERCD) and satisfaction.

## Methods: Participants, interventions and outcomes

### Study setting {9}

The study will be conducted in a medical center in northern Taiwan, with 1300 annual births and a CS rate of 29.71%. The medical center offers a 24-h system of designated obstetric coverage. https://www.vghtpe.gov.tw/Index.action.

### Eligibility criteria {10}

Using purposive sampling, the selection criteria includes women aged between 20 and 45; have experienced one previous CD; 14–16 weeks’ gestation with at least a half year interval between current pregnancy and the previous birth; and are fluent in English or Mandarin. Exclusion criteria includes women with multiple pregnancy, previous classic CD, or myomectomy, and/or pregnancy with high risk of complications (for example, women who had risk factors such as hypertension, heart disease, diabetes, epilepsy, threatened premature labor, or another preexisting medical problem).

### Who will take informed consent? {26a}

The principal researcher SWC will invite eligible women to participate in the study. Prior to the study commencement, the participants will give written informed consent for participation after the principal researcher explain the purpose and procedure of the study.

### Additional consent provisions for collection and use of participant data and biological specimens {26b}

This trial does not involve collecting biological specimens.

### Interventions

#### Explanation for the choice of comparators {6b}

The control group condition is essentially ‘usual care’ and will enable comparison with the experimental condition in regard to the shared decision making intervention only, thereby minimizing the potential impact of any additional variables in the experiment. A maternal health booklet, produced by Health Promotion Administration, Ministry of Health and Welfare, Executive Yuan, Taiwan, will be provided to all women in the study [35]. The 58-page booklet is routinely provided to all women during pregnancy in Taiwan and includes Five (5) “Dos” and “Do nots” during pregnancy. Five (5) “Dos” content includes (1) Do receive prenatal exams according to schedule; (2) Do take good care during pregnancy; (3) Do recognize pregnancy complications; (4) Do know the signs of premature birth; (5) Do know the signs of labor. Five (5) “Do nots” content includes (1) Do not smoke or drink; (2) Do not be exposed to secondhand smoke; (3) Do not take medication without doctor’s orders; (4) Do not choose a C-section to pick the time of birth; (5) Do not use drugs [35]. There is very limited information relevant to mode of birth choice after caesarean.

### Intervention description {11a}

**Fig. 2:**
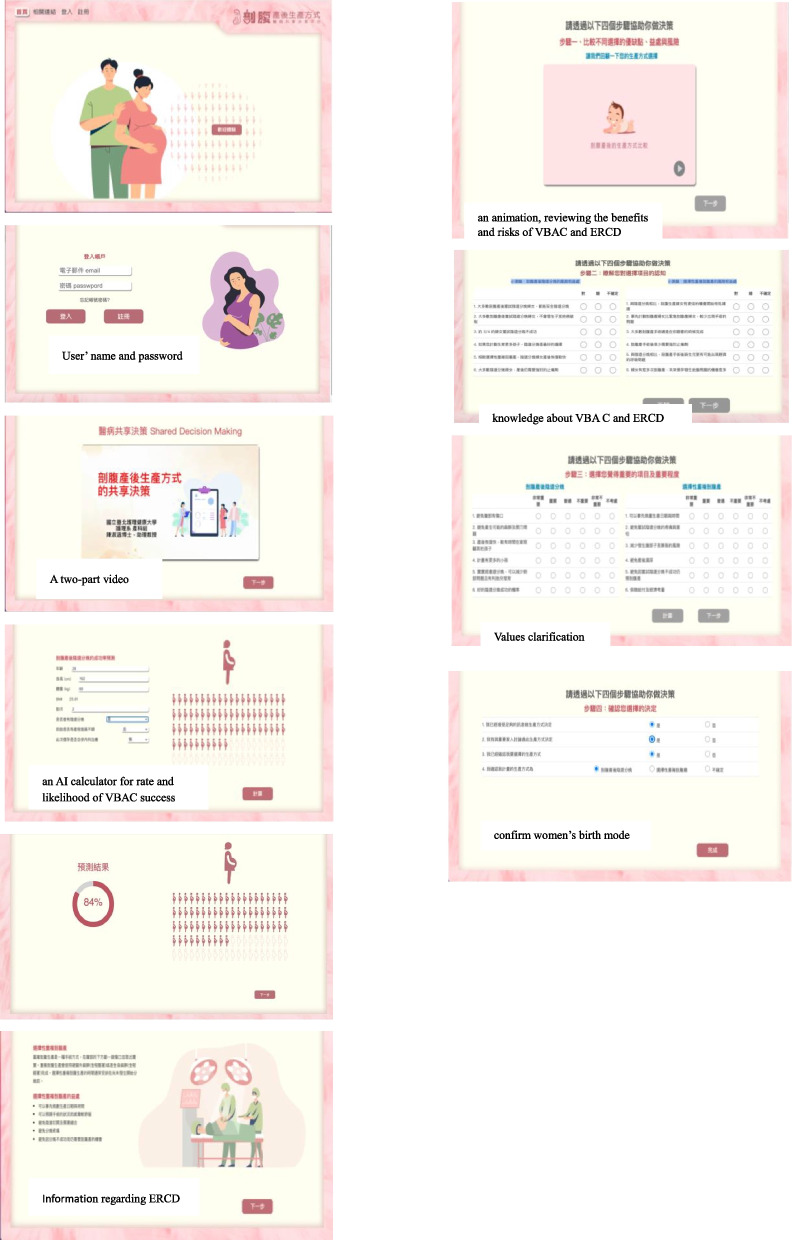
Web-based decision-aid platform

Women in the intervention group will receive both usual care plus access to the web-based decision-aid and access to on-line support. The web-based decision-aid platform was adapted from the original Birth Choices decision-aid by Shorten et al. [36, 37]. We invited 10 experts to evaluate the decision-aid platform for use in Taiwan, including five clinicians (two obstetricians, one head nurse and two supervisor), three midwives, and two academic professors. They provide feedback after consultation; the content validity was 0.97. The web-based decision-aid consists of five sections (Fig. 2). Based on the previous pilot study findings [35], we developed the platform to ensure women complete section and corresponding steps in sequence rather than skipping decision steps. Section 1 provides a two-part video, consisting of two 10-min segments. Part 1 introduces SDM, addressing how to prepare and participate in the study, and how to communicate with the obstetric provider using three talks (team talk, option talk and decision talk). Part 2 provides information with examples to practice communication skills using a BRAIN framework (benefits, risks, alternatives, intuition, and nothing) decision for a better birth plan [38]. Section 2 presents an overview of functions and features of the birth decision-aid. Section 3 presents relevant VBAC information, including definitions, benefits and risks, and an AI calculator for rate and likelihood of VBAC success [20, 33]. In this section the medical history data for pregnant women using the decision-aid platform will be retrieved from the hospital data Health Information System (HIS) system and transferred securely to the warehouse database through the local server. Simultaneously, using the AI model, the pregnant women’s clinical characteristics entered from the Web page will be integrated, cleaned, and converted into a format that will be used by AI edge computing to predict a personal success rate for vaginal birth. Finally, the results are returned to the database and displayed on the Web page through the RESTful API for the clinical staff to access. Section 4 presents the information regarding ERCD, involving definitions, benefits, and risks. Section 5 comprises four steps of decision making to meet women’s values and preferences. Step 1 consists of an animation, reviewing the benefits and risks of VBAC and ERCD. Step 2 consists of a 12-question quiz designed to measure knowledge about VBAC and ERCD. Step 3 presents 12 values clarification statements (6 for VBAC and 6 for ERCD). Using a 6-point Likert scale for each statement, women will be asked to respond to the question ‘what is important for you’, from very important (6 points) to do not consider (1 point). A score in accordance with women’s values for each option will be calculated immediately. Step 4 is designed to confirm women’s birth mode decision based on the values clarification exercise. The principal researcher will offer 2–3 individual online consultations via Line App to answer questions about the decision-aid and clarify information within the decision-aid. Women will be directed back to their provider for questions specific to their pregnancy and decision making.

### Criteria for discontinuing or modifying allocated interventions {11b}

During the trial, if the intervention group participants experience any emergent situation such as vaginal bleeding, foetal heartbeat complications etc., we will inform them of their allocation to the intervention and allow them to decide whether continue to participate in the study.

### Strategies to improve adherence to interventions {11c}

The principal researcher will offer 2–3 individual online consultations via Line App to answer questions about the decision-aid and clarify information within the decision-aid. Women will be directed back to their provider for questions specific to their pregnancy and decision making.

### Relevant concomitant care permitted or prohibited during the trial {11d}

Since there is no invasive intervention involved in this study, there will be no physiological risks involved in this study. Thus, no concomitant care and interventions are prohibited. The only psychological risk is the emotional distress that may arise from the sensitive questions asked during the counseling process. This study will provide personal assistance or referral to the relevant counseling department or support groups.

### Provisions for post-trial care {30}

There is no anticipated harm and compensation for trial participation.

### Outcomes {12}

Primary outcome measures include decisional conflict (time frame: 14–16-weeks’ gestation and 35–38 weeks’ gestation), knowledge (time frame: 14–16-weeks’ gestation and 35–38 weeks’ gestation), birth mode preference (time frame: 14–16-weeks’ gestation and 35–38 weeks’ gestation), and decision-aid acceptability (time frame: 35–38-weeks’ gestation).Secondary outcome measures include actual birth outcome (time frame: one month after birth) and satisfaction with decision scale (SWD) (time frame: one month after birth).

### Participant timeline {13}

**Fig. 3:**
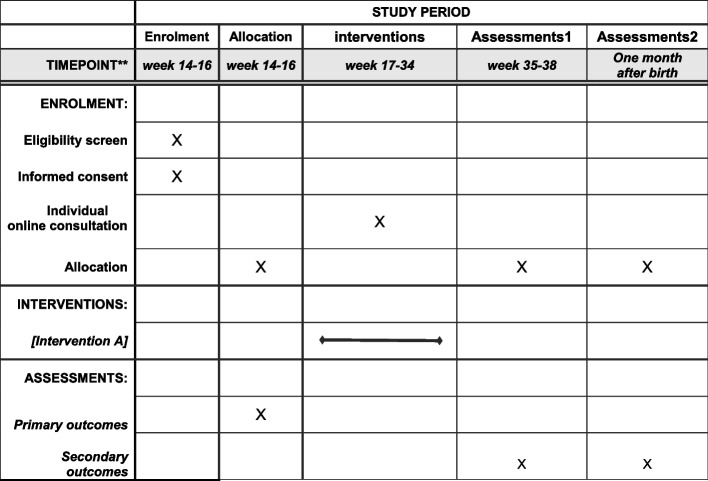
SPIRIT time schedule of enrolment, interventions, and assessments

Eligible women will be invited to participate in the study when they attend prenatal examination at the 14–16 weeks’ gestation visit in the Outpatient Department of Obstetrics and Gynecology within the hospital study site. After participants sign written consent for participation, they will be randomly allocated to either the control group or intervention group by the principal researcher. All participants will initially complete an online google survey including ‘personal demographic information’, ‘decision conflict scale’ ‘knowledge scale’ and ‘birth mode preference’. After completing the questionnaires, women allocated to the intervention group and the principal researcher will provide women a username and password to log into the birth decision-aid platform. The principal researcher will encourage women to discuss information with their families. They will be encouraged to use the decision-aid during recruitment and at each visit in the hospital Outpatient Department of Obstetrics. To answer questions and clarify information about the decision-aid, an online individual consultation with the principal researcher will be provided for women in the intervention group at least twice during the 17–34 weeks’ gestation. A first-time follow up survey will be held at 35–38 weeks’ gestation, including decision conflict scale, knowledge, birth mode preference, and decision-aid acceptability. All participants confirm preferred birth mode with their obstetrician prior to completing the first-time follow up. A second-time follow up is held one month after birth. Postnatal women will complete a “satisfaction” questionnaire when they attend their postnatal examination visit (Fig. 3).

### Sample size {14}

Sample size was calculated using G-POWER 3.1.9.2 software. Based on estimates from a previous pilot study [39], effect size = 0.50, a total of sample size 128 provided α = 0.05 and power = 0.80. To allow for preterm deliveries, mal-presentations, and losses to follow-up, an increase in the desired number by 20 percent would be 154.

### Recruitment {15}

Participants will be recruited from the Outpatient Department of Obstetrics and Gynaecology.

## Assignment of interventions: allocation

### Sequence generation {16a}

Using computer permuted block randomization.

### Concealment mechanism {16b}

A parallel group of participants will be allocated to either control group (usual care) or intervention group (usual care plus the birth decision-aid platform) in a quiet examination room in accordance with sequentially numbered computer permuted block randomization.

### Implementation {16c}

The principal research SWC will generate the allocation sequence, enrol participants, and assign participants to either control group or intervention group.

## Assignment of interventions: Blinding

### Who will be blinded {17a}

Participants will be blinded to their allocation after assignment to interventions. Data entry and analysis will both be blinded.

### Procedure for unblinding if needed {17b}

During the trial, if the intervention group participants occur any emergent situation such as vaginal bleeding, fetal heartbeat complication etc., we will inform them and allow them to decide whether continue to participate in the study.

## Data collection and management

### Plans for assessment and collection of outcomes {18a}

**Fig. 4:**
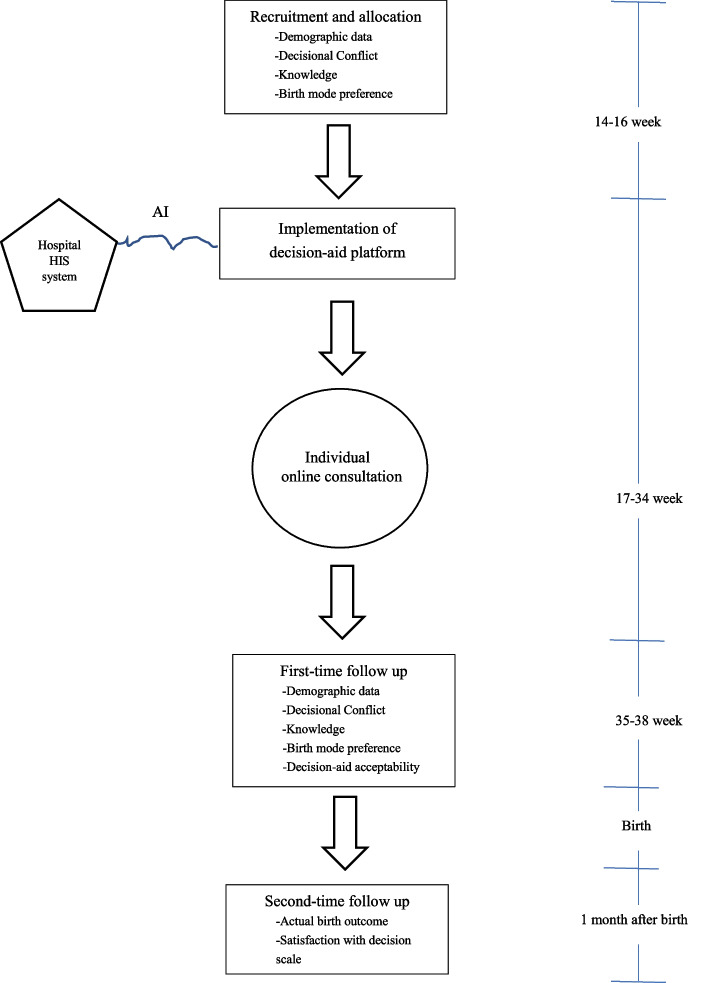
Study protocol

The principal researcher and an assistant researcher will perform data collection. Prior to the study commencing, the research team conducted an online discussion to clarify the research protocol (Fig. 4).

### Primary outcome measures

#### Decisional Conflict

The DCS was originally developed by O’Connor of the University of Ottawa [40] and was revised for this specific decision by Shorten [41]. It consists of 18 questions in five subscales measuring; certainty, informed, values, supported, and quality. A 5-point Likert-type scoring method is adopted, with each item scored from 1 to 5 points indicating strongly agree to strongly disagree, respectively. An average score <2.0 indicates readiness for decision-making. Higher scores indicate higher degrees of decision conflict. In Japan, Cronbach’s α of DCS is 0.86 [42].

### Knowledge

Knowledge measurement consists of a 15-item questionnaire which presents a series of statements regarding risks and benefits of each birth mode [41]; answering ‘true,’ ‘false,’ or ‘unsure’. Negative scoring is applied, awarding a score of + 1 for a correct answer’, -1 for an incorrect answer and zero for an “unsure” response. Previously tested Cronbach *α* for this questionnaire was = 0.67 [41].

### Birth mode preference

Women’s birth mode preference is assessed on a 15-point scale with anchor points for VBAC and ERCD. Birth preference will be measured and recorded using the 15-point scale as a rating of strength of women’s birth method preference [41].

### Decision-aid acceptability

Decision-aid acceptability includes nine questions to measure women’s rating of the birth decision-aid platform using a 5-point Likert scale ranging from ‘not at all’ (1 point) to ‘a great deal’ (5 points) [37].

### Secondary outcome measures

#### Actual birth outcome

The principal researcher will ask women about their actual birth outcome one month after birth via Line App and verify this with the medical record.

### Satisfaction with decision scale (SWD)

SWD is assessed using six questions to measure women’s satisfaction with their decision. The 5-point Likert scale ranges from “strongly agree” (5 points) to “strongly disagree” (1 point). Women have higher scores indicating increased satisfaction. SWD [43]. Cronbach’s α previously tested was 0.88 [44].

### Plans to promote participant retention and complete follow-up {18b}

Reminders will be sent to participants through the Line app to complete questionnaire at three scheduled times (14–16-weeks’ gestation and 35–38 weeks’ gestation and one month after birth). Eligible participants will receive up to $300 Taiwanese (NTD) compensation for participating one month after birth. The research team will have weekly meetings to monitor the adherence and retention of participants.

### Data management {19}

The encrypted data will be stored securely for six years in the principal researcher’s password protected computer in a locked office. Data will be maintained in a secure database that only the study investigators will have access to the final trial dataset and disclosure of contractual agreements.

### Confidentiality {27}

As per institutional approval, the data will be encrypted. Each participant will be identified by alphabetical codes (A, B, C, etc.).

### Plans for collection, laboratory evaluation and storage of biological specimens for genetic or molecular analysis in this trial/future use {33}

There will be no biological specimens collected.

## Statistical methods

### Statistical methods for primary and secondary outcomes {20a}

Analysis will be by intention to treat. Data will be processed and analyzed using IBM SPSS version 28.0 software. Descriptive statistical analysis will include frequency, percentage, mean and standard deviation for each variable; chi-square test and Student’s t-test will be used to compare the differences between groups for the homogeneity of basic attributes. Statistical significance will be established using an α of 0.05. Bivariate analyses and linear regression models will be used to examine determinants of primary outcomes (decisional conflict score, knowledge score, birth mode preference), and secondary outcomes (mode of birth and satisfaction with decision). The strength of women’s birth mode preference will be analyzed using a non-parametric test (Wilcoxon signed-rank test).

### Interim analyses {21b}

No interim analyses are planned.

### Methods for additional analyses (e.g. subgroup analyses) {20b}

Analysis of decision aid acceptability subgroup will be conducted to assess the differences between control and intervention group.

### Methods in analysis to handle protocol non-adherence and any statistical methods to handle missing data {20c}

Intention to treat analysis will be used and complete case analysis will be managed for missing data. We will undertake analysis to determine any systematic differences between those who do not adhere to the protocol or who drop out of the study.

### Plans to give access to the full protocol, participant level-data and statistical code {31c}

The datasets analysed during the current study and statistical code are available from the corresponding author on reasonable request, as is the full protocol.

## Oversight and monitoring

### Composition of the coordinating centre and trial steering committee {5d}

Dr. Shu Wen Chen as the primary investigators, will be responsible for the overall management of the project and will lead platform development and content. Dr. Allison Shorten has expertise in program development and clinical trials and will lead in the preparation and data analysis. Dr. Chang Ching Yeh is a clinical obstetrician with expertise in Obstetrics and Gynecology and will lead shared decision making for participants. Dr. Chien Huei Ka has expertise in midwifery and will develop platform. Dr. Yu Ying Lu has expertise in maternal care and will develop platform. Dr. Hsiang Wei Hu has expertise in biomedical engineering and will be responsible for AI program development.

### Composition of the data monitoring committee, its role and reporting structure {21a}

Study investigators serve as the trial coordinating group and will monitor the trial. There will be weekly meetings with clinical obstetrician, research assistants and the primary investigators (i.e., Dr. Chen and Dr. Yeh) to discuss and review each phase of the trial (e.g., recruitment, randomization, and data collection). The current trial does not require a separate data monitoring committee because of the determination of minimal risk.

### Adverse event reporting and harms {22}

Any SAEs that are classed as related and unexpected will be reported to the Human Research Ethics Committee.

### Frequency and plans for auditing trial conduct {23}

The Human Research Ethics Committee will schedule an annual audit with the Trial Steering Group.

### Plans for communicating important protocol amendments to relevant parties (e.g. trial participants, ethical committees) {25}

Any amendments to the protocol will be submitted to the Human Research Ethics Committee of Taipei Veterans General Hospital, who will review and approve the amendments. If the amendment requires that revised information be communicated with participants, this will be done through a consent addendum, which will be provided to participants through Line App.

### Dissemination plans {31a}

Research findings will be disseminated via national and international conferences. Papers will be posted rapidly on preprint servers and where possible will be open access to allow for wider reach. The publication plan includes two academic, and one non-academic journal within two years from results being available. The web-based decision-aid will be shared in several social media pages. To provide access easily for pregnant women, we intend to share the technology associated with the web-based decision-aid with government health departments such as Health Promotion Administration, Ministry of Health and Welfare or Joint Commission of Taiwan.

## Discussion

Although SDM is recommended for birth after cesarean, implementation of the innovative interactive web-based Birth Choices decision-aid for SDM may encounter several obstacles in Taiwan. Based on our previous pilot study findings, the birth choices decision-aid was found to be acceptable feasible to increase women’s knowledge, decrease decisional conflict and improve satisfaction [30]. However, Taiwanese women were not expecting to engage in SDM and did not prepare for SDM. Women were not confident in communicating with their obstetrician and were also concerned about time constraints during consultation [30]. Previous literature showed that the use of conversation or decision-aids for use within the clinical encounter, appear to promote patient-clinician interactions consistent with SDM [45]. Compared to patient decision-aids focusing on increasing knowledge and reducing decision conflict, encounter tools aim to improve the quality of the SDM process [46]. To improve the decision-making process and communication relationships within this context, aids to specifically improve women’s communication skills for decision making were designed and placed at the beginning of the decision-aid as the first engaging activity. This additional component aims to better prepare women for SDM by providing specific examples regarding how to interact with their obstetrician and preparing them to actively participate in decision making about their birth. Additionally, several previous studies found that women were reluctant to choose trial of labor because of lacking confidence in their ability to achieve VBAC [29, 30, 31, 34].

The innovative web-based decision-aid platform combining communication education and birth decision-aid is designed to improve the decision-making process and relationships between women and obstetric providers in Taiwan, with potential to improve the SDM experience and VBAC rate. Additionally, the platform may support change in provider intention towards shared decisions with women and families as it integrates decision-aids within clinic workflow. Particularly web-based data analysis using AI linked to the medical record, may be beneficial for supporting SDM in clinical practice.

## Trial status

We began to recruit participants from September 15, 2021, based on the protocol October *2021 (v1)*. Because of the COVID-19 pandemic, recruitment of participants at the hospital was paused. Estimated primary completion date will be September 31, 2023.

## Data Availability

Any data required to support the protocol can be supplied on request.

## Abbreviations

CD: Caesarean Delivery
ERCD: Elective Repeat Caesarean Delivery
VBAC: Vaginal Birth after Caesarean
SDM: Shared Decision Making
